# Nutrition in Patients with Lactose Malabsorption, Celiac Disease, and Related Disorders

**DOI:** 10.3390/nu14010002

**Published:** 2021-12-21

**Authors:** Michele J. Alkalay

**Affiliations:** Department of Pediatrics, Division of Pediatric Gastroenterology, Hepatology and Nutrition, University of Texas Southwestern, Dallas, TX 75235, USA; MicheleJ.Alkalay@UTSouthwestern.edu; Tel.: +001-469-497-2505

**Keywords:** lactose malabsorption, lactose intolerance, celiac disease, gluten-related disorders, FODMAPs, nutrition, diet adherence, gluten-free diet, non-celiac gluten sensitivity, irritable bowel syndrome

## Abstract

Lactose malabsorption (LM), celiac disease (CD), non-celiac gluten sensitivity (NCGS), and irritable bowel syndrome (IBS) are conditions associated with food triggers, improvement after withdrawal, treatment with dietary restriction, and subsequent nutritional detriments. LM occurs when there is incomplete hydrolysis of lactose due to lactase deficiency and frequently produces abdominal symptoms; therefore, it can cause lactose intolerance (LI). A lactose-restricted diet is frequently recommended, although it can potentially lead to nutrient deficiencies. Furthermore, lactose is an essential component of fermentable oligo-, di-, and monosaccharides and polyols (FODMAPs) and is subsequently associated with intolerance to these compounds, especially in IBS. LM commonly presents in CD. Nutritional deficits are common in CD and can continue even on a gluten-free diet (GFD). Conditions triggered by gluten are known as gluten-related disorders (GRDs), including CD, wheat allergy, and NCGS. IBS can also be associated with a gluten sensitivity. A GFD is the treatment for CD, GRDs, and gluten sensitive IBS, although compliance with this restricted diet can be difficult. Strict dietary therapies can have a negative effect on quality of life. This review aims to provide an overview of the difficult nutritional elements of these disorders, which are critical for medical providers to recognize when managing these patients.

## 1. Introduction

Lactose malabsorption (LM), celiac disease (CD), non-celiac gluten sensitivity (NCGS), and irritable bowel syndrome (IBS) are disorders characterized by the onset of symptoms from ingestion of a particular food and by relief after its elimination from the diet. Consequently, patients with these disorders are placed on restricted diets. However, this can lead to significant nutritional deficiencies. Lactose is a disaccharide that comprises the monosaccharides glucose and galactose, the primary carbohydrate found in mammalian milk [1]. LM is caused by the incomplete hydrolysis of lactose due to lactase deficiency, the reduced expression of lactase enzyme in the small intestine. LM may occur as a primary or a secondary disorder due to other intestinal diseases. LM leads to lactose intolerance (LI), the occurrence of gastrointestinal symptoms post-ingestion of lactose. A lactose-restricted diet is typically recommended for symptom relief, although it may lead to nutritional disadvantages with reduced calcium and vitamin intake. The frequency of LI varies according to ethnicity and has been reported as high as almost 100% in Southeast Asia, approximately 80% in Southern Europe, and <5% in Northern Europe [2]. Furthermore, lactose is an essential element of fermentable oligo-, di-, and monosaccharides and polyols (FODMAPs) and is subsequently correlated to the broader intolerance of the absorption of these short chain carbohydrates. FODMAPs can cause digestive discomfort, which has been commonly reported in IBS patients. A low FODMAPs diet has been shown to improve IBS symptoms in 50–86% of patients. However, this diet is very restricted and risks nutritional insufficiency and encourages disordered eating [3]. 

Furthermore, CD is a chronic autoimmune intestinal disorder triggered by gluten consumption in genetically predisposed people, affecting 1% of the general population worldwide. The ingestion of gluten in CD causes an inflammatory response with resultant damage to the small intestine. LM occurs in CD due to the loss of lactase enzyme on the damaged small intestinal villi [4]. LI occurs frequently in CD, estimated to be 10% and increasing up to 50% in the presence of malabsorption [5]. Nutritional deficiencies occur frequently in CD. During early diagnosis, at least one nutrient deficiency has been reported in almost 90% of untreated adult celiac patients [6]. Nutrient deficits can persist despite being placed on a gluten-free diet (GFD) [7,8]. Diseases caused by ingestion of gluten are called gluten-related disorders (GRDs), which include CD, wheat allergy, and NCGS. GRDs have an estimated 5% global prevalence [9]. In addition, approximately one-third of IBS patients present with gluten sensitivity [10]. A GFD is the only effective treatment for CD, GRDs, and IBS gluten sensitivity; however, adherence to the strict GFD can be problematic. Furthermore, the restricted diet used to treat individuals with LM, GRDs, and IBS can negatively impact their daily lives. Effects of nutrition on the health of patients with these conditions are challenging and are important for health-care providers to recognize when managing these diseases.

The aim of this review is to shed light on the nutritional aspects of LI, CD, NCGS, and IBS with an overview of these diseases and emphasis on practical issues in the management of patients. Treatment of these conditions involves dietary restriction; however, it is crucial for clinicians to be aware that restricted diets can cause nutrient deficits, affect quality of life, and pose difficulties with compliance. Disorders of lactose, gluten, and irritable bowel currently present with evolving diagnostic measures, significant nutritional disadvantages, advancing methods in measuring diet adherence, and rising challenges in the long-term medical care of these patients.

## 2. Lactose Malabsorption and Intolerance

Absorption of lactose requires lactase enzyme activity in the small intestinal brush border to split the two bound monosaccharides. Lactase is found in the small intestine on the tips of the villi [1]. When there is low lactase enzyme activity, lactose absorption is consequently reduced. LM is the imbalance between the amount of lactose ingested and the ability for lactase to hydrolyze the disaccharide. If lactase enzymes are absent or deficient, then unabsorbed lactose in the small intestine produces an osmotic load with resultant secretion of fluid and electrolytes into the lumen. This also leads to the fermentation of undigested lactose by the colonic microbiome, triggering the release of hydrogen, carbon dioxide, and methane. The increase in osmotic load and gas production in LM can cause gastrointestinal complaints [11]. LI is the clinical syndrome after lactose ingestion, including one or more of the following symptoms: abdominal pain, diarrhea, nausea, flatulence, and/or abdominal bloating. Primary lactase deficiency is the absence of lactase and is the most common cause of LI. Secondary lactase deficiency is due to small bowel injury, such as gastroenteritis, CD, Crohn’s disease, or chemotherapy [12]. As much as 70% of the world’s population have been estimated to have primary lactase deficiency [13]. The amount of lactose consumed that causes symptoms is variable from one individual to another and is dependent on several factors, including the amount of lactose consumed, lactase expression, intestinal transit time, small bowel bacterial overgrowth, and the composition of the enteric microbiome [14].

As LI is a clinical condition with symptoms post-ingestion of lactose, it can be diagnosed clinically and/or by various other methods. The hydrogen breath test (HBT) involves the oral administration of lactose (usually 25 grams) and the measurement of the quantity of exhaled hydrogen (recollected every 30 min after oral administration of lactose). The HBT is positive when the hydrogen level is at least 20 parts per million greater than the baseline value in the exhaled air. This study is considered the gold standard for diagnosis of LI, with high sensitivity and specificity [15]. Testing lactase enzyme activity in mucosal duodenal biopsies is considered the reference standard for lactase deficiency; however, it has limitations in the expression of lactase enzyme on the small intestinal villi and the invasiveness of the test [16]. Genetic tests may be helpful in identifying groups of people with lactase non-persistence [17,18,19]; however, genes and biopsies measuring lactase enzyme do not address the clinical symptoms that lactose intolerant patients experience.

The treatment for LM and LI is a lactose-restricted diet, reducing the dietetic amount of lactose until clinical symptoms disappear. Most lactose intolerant individuals can tolerate less than 12 grams of lactose per single dose ingested in the diet [20,21]. However, many countries do not have laws regulating commercialization of lactose-free products, which poses the problem of mislabeling and causation of symptoms in lactose intolerant individuals [22]. Furthermore, lactase enzyme replacement may be helpful in patients with isolated LI when ingesting dairy products. Exogenous lactase breaks down lactose into glucose and galactose to enable better absorption [23]. However, the lactase enzyme may not completely relieve symptoms due to the incomplete digestion of lactose; therefore, enzyme supplementation should not be the sole treatment for LI. Lactase enzyme should rather be supplementary to dietary restriction [24]. Furthermore, studies suggest that altering the intestinal microbiota by probiotic supplementation in lactose intolerant patients can alleviate symptoms. A clinical trial involving probiotics (DDS-1 strain of lactobacillus) in LI patients versus a placebo discovered that it decreased symptoms, including diarrhea, abdominal pain, and emesis [25]. Another study used a mixture of probiotics, Bio-25, lactase-producing bacteria that showed significant alleviation of gastrointestinal complaints associated with LI; however, there was no reduction in hydrogen excretion to HBT of these patients [26]. Systematic reviews have shown that probiotics have an overall positive effect on LI [27,28]. As probiotics have no side effects and improve clinical symptoms, they should be considered for the treatment of LI patients. Prebiotics have also been considered in the treatment of LI in a few studies; however, further analyses are needed to investigate the benefits in clinical trials that gather more evidence [29,30,31].

## 3. Lactose-Restricted Diet and Nutritional Disadvantages

Restricting lactose in the diet may reduce gastrointestinal complaints, although it can predispose patients to nutritional risks. Dairy products are valuable sources of calcium, Vitamin D, protein, and other minerals, including, magnesium, potassium, phosphorus, and zinc. They can have positive effects on bone health, such as bone remodeling [32]. Although LM does not cause calcium malabsorption, dietary avoidance of milk products can lead to sub-optimal bone mineralization [33].

During childhood, dairy products have been shown to be important for bone growth and bone health. There is evidence of significant advances in height, body weight, bone mineral content, and bone mineral density in children who consume dairy products [34,35,36]. Children who avoid milk products have been documented to ingest less than the recommended amounts of calcium needed for normal bone growth and bone mineralization [37]. Avoiding milk has been linked to a lower bone mineral content and increased fracture risk in children [38]. It is important to note that the association between LI and osteoporosis remains unclear [39]. Some studies have shown a positive relationship between LI, low bone mineral density, and osteoporosis [40,41]. Other studies have shown no significant association between LI and bone mineral density [42,43].

Further studies are needed to account for LM and long-term health outcomes. However, if dairy products are eliminated, it is recommended that calcium intake from other foods be increased or that calcium supplements be provided. Individuals with LI should restrict lactose instead of avoiding lactose, so as not to lose the nutritional value of dairy products in the diet. Therefore, consideration should be given to a temporary lactose-free diet with the goal of reducing symptoms, followed by a gradual introduction of dairy products as tolerated [44].

## 4. Celiac Disease

CD is a common autoimmune enteropathy in genetically susceptible individuals, precipitated by the consumption of gluten, the protein found in wheat, rye, and barley. The immune-mediated reaction causes villous atrophy of the small intestine, specifically in the duodenum, with subsequent malabsorption. There is complete resolution of CD on a GFD; however, the duodenal inflammation relapses when gluten is reintroduced. Regarding genetic susceptibility, 99% of celiac patients carry haplotype HLA DR3-DQ2 and/or DR4-DQ8, in comparison to approximately 40% of the general population [45]. CD is usually detected by serologic testing of celiac specific antibodies, tissue transglutaminase (TTG), endomysial antibody (EMA), and deamidated gliadin peptide (DGP), and the diagnosis is confirmed by endoscopic duodenal mucosal biopsies when villous atrophy is present [46,47]. Though antigliadin antibodies (AGAs) are prevalent in celiac patients, they are not specific and have been replaced by the more specific antibodies (TTG, EMA, and DGP) to detect CD. Small intestinal biopsy historically has been the gold standard for diagnosis; however, a serology-based diagnosis for CD with the omission of endoscopic biopsies has been an accepted approach in selected pediatric cases [48,49]. The treatment for CD is a life-long GFD; the small intestinal damage recurs when gluten is re-ingested.

## 5. Nutritional Deficiencies in Celiac Disease

In untreated CD, the small intestinal villi atrophy on the apical enterocyte brush border where lactase normally resides. This causes a partial deficiency in lactase enzyme, with resultant LM and secondary LI. Lactase deficiency has been shown to be common amongst celiac patients, and LI can be the sole initial manifestation of undiagnosed CD. There is evidence that 24% of patients with LI determined by HBT have CD, diagnosed by positive serology and biopsy [5]. LI symptoms can overlap with those of CD, including abdominal pain, bloating, and diarrhea. Not only do LI and CD share common clinical presentations, they also have a response to food withdrawal [4,21]. In fact, clinicians tend to over-diagnose patients with LI and can possibly miss the diagnosis of CD, especially in the pediatric population [50,51,52]. Once being placed on a GFD for CD treatment and following the restoration of the mucosa, most patients can tolerate lactose one to two months later [12]. HBT has been suggested for monitoring mucosal healing in treated CD individuals [53]. However, LI has been frequently reported in CD patients who are considered unresponsive to a GFD. Disaccharidase deficiency, specifically lactase, has been notable in these individuals when biopsied. Nonresponsive celiac disease (NRCD) has been described as occurring in approximately 20% of celiac patients. LI is a common cause, reported in up to 8% of NRCD patients [54]. These individuals may require a long-term lactose-restricted diet.

Due to the damage of the small intestinal villi that are important in the absorption of micro- and macronutrients, the consequence of CD is predominantly malnutrition secondary to malabsorption. Pediatric patients can present with failure to thrive, due to chronic malabsorption. As there is an increased gut permeability from the autoimmune inflammatory response in CD, this can lead to symptoms of diarrhea, steatorrhea, weight loss, anemia, and even osteopenia [46]. CD is often a cause of low bone density, with an increased fracture risk in celiac patients. This is due to impaired absorption of vitamin D and calcium, likely resulting in hyperparathyroidism. During initial presentation of CD, vitamin D levels are often lower and parathyroid levels are often higher than in healthy controls [55,56,57]. Reduced bone mineral density, including osteopenia and osteoporosis, occur in approximately 70% of celiac patients at diagnosis [58]. Common nutritional deficiencies in newly diagnosed and untreated CD are iron, B12, calcium, Vitamin D, zinc, and copper [8,59]. In most patients diagnosed with CD, a strict GFD should result in complete resolution of their symptoms of the disease. However, despite a GFD, nutritional deficiencies can persist in approximately 30% of CD patients [60,61,62,63].

## 6. Irritable Bowel Syndrome

IBS is a functional bowel disorder characterized by chronic symptoms of abdominal pain, changes in bowel habits, and bloating without a known organic cause. IBS affects greater than 5% of the general population and is the most prevalent of functional gastrointestinal disorders. Diagnosis for IBS patients is based on their clinical assessment using the Rome IV criteria (i.e., recurrent abdominal pain associated with defecation or a change in bowel habits with symptom onset at least 6 months prior to diagnosis and present for the last 3 months). IBS is classified into three main subtypes according to the bowel pattern: IBS-C (constipation-predominant), IBS-D (diarrhea-predominant), and IBS-M (mixed bowel habits) [64]. The exact pathophysiology of IBS is not well understood, although proposed mechanisms include alterations in visceral hypersensitivity, disordered gut-brain interaction, intestinal microbiome, enteric sensory and motor dysfunction, altered pain processing, and immune dysregulation [65]. Diet plays a key role in IBS and has been reported to be a symptom trigger in approximately 80% of patients. Individuals with more severe IBS identify a greater number of food items as stimuli for their gastrointestinal symptoms [66]. Subsequently, there is evidence for the utilization of dietary therapies in IBS. Traditional dietary advice of healthy eating habits has been implemented to treat IBS patients, and incorporates lifestyle management, eating regular meals, lowering fat consumption, adequate liquid intake, and assessing the intake of fiber, alcohol, caffeine, and spicy foods [67]. Two specific IBS dietary interventions that have been studied in several randomized controlled trials are low FODMAPs diets and the GFD [68].

## 7. Nutritional Aspects of Irritable Bowel Syndrome, FODMAPs, and Gluten Sensitivity

Patients can have an intolerance to FODMAPs, which includes lactose, with symptom improvement when placed on a low FODMAPs diet [69,70,71]. FODMAPs are short-chained carbohydrates that are not well absorbed by the gastrointestinal tract, due to their increased osmotic load and resultant excess gas production from bacterial fermentation. A low FODMAPs diet is a 2-phase intervention, with strict avoidance of short-chain carbohydrates followed by reintroduction of specific tolerated FODMAPs. Some studies show an improvement in the quality of life in IBS patients on a low FODMAPs diet [71,72,73]. In contrast, other studies have shown a negative impact on quality of life due to strict dietary restriction. A low FODMAPs diet may affect sleep patterns, energy, costliness, and social functioning, with an increased risk of disordered eating behaviors [66,74,75]. Disordered eating patterns include skipping meals, binge eating, restricting certain foods, or fasting, a deviation from the cultural standard of eating three meals a day. These behaviors have been linked to orthorexia anorexia, an obsession focused on food choices, planning, preparation, and consumption with the loss of pleasure when eating, affecting an individual’s daily well-being [74]. 

Additional long-term studies assessing the quality of life in IBS patients when placed on a low FODMAPs diet are necessary. It is important for healthcare providers to understand that any restrictive diet can lead to malnutrition. Certain nutrients are at risk for depletion in a restricted FODMAPs diet, including fiber, calcium, protein, iron, and Vitamin B12 [75,76]. Therefore, patients who are placed on a low FODMAPs diet should be educated by a trained dietician to avoid nutritional depletion. Furthermore, there are several studies that show the change in the gastrointestinal microbiota on a low FODMAPs diet [77,78,79,80]; however, the exact implications on long-term health have yet to be discovered.

In addition to the intolerance of FODMAPs, many IBS patients suffer from gluten sensitivity. Gluten has been reported to generate symptoms in up to 30% of individuals with IBS [10]. Studies have demonstrated the improvement of IBS symptoms in individuals who are on a GFD, such as constipation, abdominal pain, and especially diarrhea [81,82,83]. There is some evidence that small bowel permeability increases after a gluten challenge in gluten sensitive IBS-D patients [81,84]. The exact mechanism is unclear and further investigations with larger populations are needed to confirm this finding. In addition, AGAs (non-specific antibodies prevalent in celiac patients) have been reported positive in IBS populations [83,85]. IBS patients with AGAs reported less diarrhea when placed on a GFD than those patients without antibodies. This indicates the potential to predict a clinical response to a GFD in IBS AGA-positive patients [83]. However, the AGA positivity of the general IBS population is unknown, and 7% of the general population has AGA present [86]. The uncertain relevance of AGA testing in IBS warrants further studies. Management of gluten sensitive IBS patients involves dietary therapy with a GFD. However, avoiding gluten in the diet can lead to micronutrient deficiency [87]. Despite being exposed to some gluten, IBS gluten sensitive patients have shown clinical improvement on a GFD [83]. Therefore, a gluten-reduced diet should be considered in lieu of a strict GFD for IBS patients, potentially leading to symptom improvement and the avoidance of nutritional complications [88].

## 8. Non-Celiac Gluten Sensitivity

NCGS is a GRD, a condition affected by the consumption of gluten. GRDs mainly involve CD, NCGS, and wheat allergy. Sometimes referred to as non-celiac wheat sensitivity, NCGS is recognized as a distinct clinical syndrome from IBS and other GRDs. These patients present similarly to celiac patients who have no signs of intestinal damage [9,89]. NCGS is a condition characterized by symptoms related to the ingestion of gluten in the absence of CD and wheat allergy. The diagnosis is often made by exclusion and confirmed by improvement of symptoms after the removal of dietary gluten. Symptoms can be gastrointestinal (bloating, abdominal pain, diarrhea, constipation, and nausea) or extraintestinal (fatigue, headache, aphthous stomatitis, arthralgia, muscle pain, depression, and reduced mental clarity). The re-introduction of gluten in the diet causes recurrence of symptoms [89,90,91,92].

Regarding the genetics of NCGS, evidence reveals that there is no correlation between NCGS and the CD predisposing haplotypes, HLA-DQ2 and/or HLA-DQ8 [89,91]. The genetic background of NCGS has not yet been revealed. Furthermore, there are histologic features in NCGS, including duodenal and colonic eosinophil infiltration [10], and duodenal biopsies with Marsh 0 (normal) to I classification (infiltration of intraepithelial lymphocytes (IELs)) without villous atrophy [91,93,94]. Potential predisposing factors for NCGS are autoimmune and functional gastrointestinal disorders, female sex, neurological disorders, eating disorders, first-degree relatives with CD, and adverse reactions to foods [95].

The pathophysiology of gluten sensitivity in non-celiac patients is not well understood. NCGS potentially involves an innate immune response activated by gluten and other wheat proteins. Wheat components other than gluten, such as FODMAPs and amylase-trypsin inhibitors, have been shown to trigger clinical manifestations of NCGS [95,96,97]. Upregulated levels of toll-like receptor 2 and downregulated levels of the T-regulatory cell marker factor forkhead box P3 have been found in intestinal biopsies of NCGS patients, in comparison to CD patients and healthy controls [98]. Studies reveal an increased expression of transforming growth factor α, interleukin 10, CD14, granulocyte-macrophage colony-stimulating factor, C-X-C motif chemokine ligand 10, and lipopolysaccharide-binding protein in NGCS patients [81,99,100]. However, the production of tumor necrosis factor and IL-17 in rectal tissue of NCGS subjects suggests an adaptive immune response [101]. AGA positivity in NCGS patients also supports the involvement of an adaptive immunopathology [90,91]. In addition to the activation of the innate and adaptive immune systems, recent evidence suggests that the pathogenesis of NCGS involves alterations at the intestinal level (inflammation, dysbiosis, and altered barrier function) and the translocation of microbial and dietary products [95,102,103,104].

There are currently no diagnostic tests for gluten sensitivity in non-celiac patients. Due to the absence of NCGS biomarkers, a double-blind placebo-controlled gluten challenge is the only method to confirm the diagnosis [10]. This test requires providing the patient with a blind administration of increasing doses of gluten and a placebo, while recording the results. The procedure is complicated and, therefore, impractical for routine clinical practice. In addition, AGA has been proposed to serve as a potential marker for NCGS. Studies on serologic testing in NCGS patients have revealed prevalent positive AGAs, particularly immunoglobulin G anti-gliadin, with negative EMA, TTG, and DGP antibodies [90]. However, AGA is not specific and is increased in both CD and gluten sensitive IBS patients. 

Interestingly, the prevalence of AGA positivity in NCGS patients has been reported as being similar to that in individuals with gluten sensitive IBS [10,83,90,91]. Furthermore, several biomarkers have been suggested for NCGS, such as T helper lymphocytes, mast cells, cytokine levels, serum antibody levels, intraepithelial CD3^+^T cells, evaluation of eosinophils, RNA transcripts, and miRNA signatures [10,105,106,107,108,109]. However, these markers are not specific for NCGS. Diagnosing NCGS without sensitive and specific markers can be challenging.

Further complicating the diagnosis, NCGS can have overlapping symptoms with IBS and other GRDs, specifically CD and wheat allergy. Obtaining a serum Immunoglobulin E (IgE) antibody against wheat protein can help distinguish between wheat allergy and gluten sensitivity. Wheat allergen specific IgE is not detected in NCGS. Furthermore, NCGS patients do not express celiac-specific autoantibodies, distinguishing the two diagnoses [89]. On duodenal biopsy, both NCGS and CD may display an increased infiltration of IELs (class I histologic Marsh classification); however, CD can only present with villous atrophy (Marsh III classification) [110]. 

Additionally, distinguishing NCGS from gluten sensitive IBS can be complex. Neither disorder has a well-known pathophysiology, making their differentiation more difficult. In addition, NCGS and gluten sensitive IBS can share a similar clinical presentation. NCGS patients commonly present with IBS symptoms, such as bowel habit changes, abdominal pain, and bloating. Over 20% of patients with self-reported NCGS fulfill the IBS Rome III criteria (i.e., abdominal pain or discomfort for at least 3 months, with onset at least 6 months prior in relation to defecation) [111]. The difference between these two conditions is that NCGS patients self-report symptoms when ingesting gluten and identify it as a symptom trigger. In contrast, IBS patients do not report gluten as a specific culprit for their symptoms. However, wheat is commonly reported as a food intolerance in IBS patients. Without sensitive markers, it can be difficult to set apart gluten sensitivity of non-celiac patients from those with IBS. Three notable differences between NCGS and IBS are the more frequent presence of extraintestinal symptoms in NCGS than in IBS, the absence of IELs in endoscopic biopsies in IBS cases, and the reaction of NCGS patients solely to gluten and not to other foods. Some NCGS patients continue to complain of symptoms despite a strict GFD, and a low FODMAPs diet can potentially alleviate their symptoms [112]. A GFD and a low FODMAPs diet can reduce symptoms in individuals with both IBS and NCGS [10], again making it difficult to separate NCGS from IBS cases. Therefore, further studies are needed to assess the relationship between GRDs and gluten sensitive IBS.

The treatment for gluten sensitivity is a GFD, together with the guidance and support of a registered dietician [113]. Currently, therapeutic intervention for NGCS is life-long avoidance of gluten. NCGS patients have been reported to have wheat as a trigger even after 8 years on a strict GFD [114]. However, it is debated whether prolonged restriction is necessary. Evidence reveals that NCGS patients have gluten tolerance thresholds [115]. Consideration should be given to a gluten rechallenge in NCGS after one year of following a strict GFD, followed by a determination of the dose of gluten that is tolerable in each patient [116]. Therefore, patients may require a gluten-reduced diet in NCGS.

## 9. The Gluten-Free Diet and Diet Adherence

The nutrient deficits observed in CD may be caused by the disease itself, with small intestinal damage and/or the consequence of a GFD. Accumulation of heavy metals has been reported in individuals on a GFD [117]. CD patients with over 2 years of good compliance with a long-term GFD have shown micronutrient deficiency in up to 30% of subjects for vitamin B12, 40% for iron, 20% for folic acid, 25% for Vitamin D, 40% for zinc oxide, 3.6% for calcium (in children), and 20% for magnesium (in children) [118]. If dietary changes are inadequate in correcting these nutrient deficiencies, supplementation may be necessary. Therefore, it is crucial for clinicians to continue to monitor for nutritional deficiencies in CD patients and to provide access to a skilled dietician for these individuals.

Celiac patients should be differentiated from those who are non-celiac with gluten sensitivity to identify the risk for complications of CD and to determine the necessary degree and duration of adherence to a GFD. Unlike NCGS, CD has the comorbidities of increased risk of osteoporosis, infertility, increased mortality, and certain types of malignancies [119]. Furthermore, there is evidence that NCGS patients eat differently than healthy individuals, and consume less protein, carbohydrate, fiber, and polyunsaturated fatty acids. Therefore, their diets should be also guided by a registered dietician to potentially correct and avoid nutritional deficits [120].

With the growing awareness and increase in reported gluten sensitivity, many non-celiac individuals are on a GFD, which can result in nutritional detriments. Studies find up to 5% of the Western population follow a GFD, and 13% self-report gluten sensitivity [110,121,122]. Some studies have shown an increased risk of obesity and metabolic syndrome on a GFD, although other studies have shown no associated risk of coronary heart disease on a GFD, and possible prevention of diabetes [123,124,125]. In addition, an increase in body mass index in children on a GFD has been observed, which may be due to improvement of nutrient absorption in CD or to the consumption of starchy gluten-free replacement foods [126]. Many studies have shown that gluten-free products can have low nutritional value. Some gluten-free products have been reported to have an increased total of saturated fat, higher glycemic index, low protein, low fiber, and lower levels of micronutrients (Vitamin D, E, B12, iron, magnesium, potassium, and sodium) [7,120,127,128]. Naturally gluten-free foods are preferred over manufactured products, as natural foods have a higher nutritional value in lipid composition, vitamin content, and energy. Leafy green vegetables, legumes, fish, and meat are rich in iron and folic acid. Amaranth, quinoa, and buckwheat are healthy gluten-free pseudocereals, alternatives to wheat, and good sources of protein, fiber, carbohydrates, and polyunsaturated fatty acids. They are also rich in vitamins, such as folic acid, riboflavin, Vitamin E and C [128].

Furthermore, the GFD changes the gut microbiome. In children, it has been reported that CD patients have a decrease in lactobacilli and an increase in enterobacteria [129]. Studies have shown a depletion in the probiotic species (*Lactobacillus* and *Bifidobacteria*) and improvement of pro-inflammatory bacteria of CD patients on a GFD. In addition, NCGS patients show depletion of beneficial species (*Bifidobacteria*) and increasing pro-inflammatory bacteria (*Enterobactericeae* and *Escherichia coli)* [130]. Further studies are needed to determine the significance of the changes in the intestinal microbiota when avoiding gluten.

Many individuals have trouble adhering to a GFD. Several factors have been reported to affect adherence to a GFD in CD. Facilitators in complying with the strict GFD include the understanding of the diet itself, membership of a CD advocacy group, the perception of the ability to maintain compliance despite stress, mood changes, and travel [131], cognitive, emotional, and socio-cultural influences, and regular follow-up with a dietician [132]. Another important determinant of adherence to a GFD in celiac patients is the presentation of classical symptoms at diagnosis, such as diarrhea and weight loss [133]. However, the factors of young age at diagnosis, adolescence, long duration of disease, non-academic education, below-average income, and no gastroenterology follow-up have been shown to be barriers to GFD adherence [134,135]. In addition, anemia and dermatitis herpetiformis have indicated poor long-term compliance [133]. Furthermore, there are positive predictors of long-term GFD compliance, including classical CD symptoms at diagnosis and upon initial adherence to the diet. Although GFD adherence changes minimally over time, the trend of change is improvement [133,135].

Approximately 40% of children with CD experience ongoing gluten exposure even after starting a GFD [136]. In Europe, pediatric adherence to a GFD is approximately 50%, and unintentional contamination is common [137]. Pediatric GFD non-compliance has been attributed to poor palatability, difficulties in dining outside the home, poor availability of gluten-free products, and asymptomatic disease diagnosed by screening [138]. In addition, pediatric patients with newly diagnosed CD have been found to have a lower quality of life compared to healthy children and to report physical and emotional impairment, with difficulties in school and social functioning [139]. Children require regular assessment by an experienced dietician for nutritional needs, and continual clinical follow-up by a pediatric gastroenterologist.

Gluten exposure is the most common cause of NRCD in adult patients, reported at a prevalence rate of up to a 50%; however, it is difficult to distinguish between purposeful versus accidental gluten exposure [140]. Up to two-thirds of adult patients claimed never to have intentionally consumed gluten. Furthermore, they also expressed negative emotions of isolation and frustration due to the restrictions of the GFD, especially with respect to difficulties related to food labelling and eating away from home; however, these negative emotions are notably experienced less often when a person is on a diet for more than five years [141]. Noncompliance with the GFD is the leading cause of failure to respond in patients with CD. 

Non-adherence to the GFD increases the risk of morbidity and mortality, including infertility, skeletal disorders, and malignancy in CD [8]. Therefore, counseling at the time of celiac diagnosis and ongoing long-term medical follow-up are essential in the care of CD patients.

The vital role of the dietician in CD is to provide proper instruction to patients on how to follow a GFD. Patients who identify themselves as having poor food knowledge are at a higher risk for noncompliance [142]. A qualified registered dietician educates celiac patients on the GFD, evaluates for nutrient depletion, and supports nutritional status at initial diagnosis and throughout the disease [143]. Similarly, an experienced dietician is recommended when treating patients with a GFD in NCGS and gluten sensitive IBS, and when placing IBS patients on a low FODMAPs diet [72]. A specialist dietician is important in educating patients when implementing strict dietary therapies, to help prevent nutritional deficiencies.

There are several methods to assess GFD adherence in patients. Clinicians can follow celiac autoantibodies for normalization in monitoring response to a GFD, although serologic tests can often take 6–24 months to decrease after gluten has been eliminated from the diet [144]. In children, the measurement of the negative TTG antibody did not correlate well with good compliance [145]. In addition, questionnaires have been developed to measure GFD adherence. The Biagi score entails four fast and simple questions to monitor GFD compliance that has been associated with both persistent villous atrophy and EMA antibodies [146]. The validated Celiac Dietary Adherence Test (CDAT) is a standardized evaluation of GFD adherence by a clinically relevant, easy survey [142,147]. The CDAT can be used as a fast tool for screening for compliance with a GFD in patients with CD. As compared to the CDAT, the Standardized Dietician Evaluation (SDE) score has been found to correlate better with serologic and histologic findings [148]. Recent studies reveal the measurement of gluten immunogenic peptide (GIP) in human samples to determine gluten ingestion. Testing GIP in both urine and stool samples has been reported to detect gluten consumption in celiac patients and was concordant with over 65% of dietary reports. Testing was performed by enzyme-linked immunosorbent assay (ELISA) for stool and point-of-care tests (POCTs) for GIP excretion in urine and stool [149]; however, the lack of standardization in urinary GIP makes it an unreliable tool to assess adherence to the GFD [150]. Measuring GIP in stool of celiac patients may be helpful for monitoring GFD compliance [151,152]. In addition, GIP in stool has been measured in gluten sensitive IBS patients and found to correlate with a decreased level when a patient is placed on a GFD. However, GIPs were found in all subjects at baseline [83]. Therefore, further studies are necessary to develop reliable GIP controls to accurately assess GFD compliance in CD and other GRDs.

Strict adherence to a rigid diet can negatively impact the quality of life for patients with food sensitivity. These negative factors are important for clinicians to be aware of when caring for these patients. Any restrictive diet, such as lactose-free, low FODMAPs, or gluten-free, can exacerbate eating disorder behaviors. Individuals can develop food aversion or anxiety regarding the preparation of their food, or avoid social situations related to eating, leading to orthorexia nervosa, a condition in which people restrict their diet based on its quality. There is evidence that patients diagnosed with CD, IBS, and inflammatory bowel disease have high prevalence rates of disordered eating practices. Individuals may develop food aversions, with the development of fear of foods being contaminated, and subsequently become too afraid and anxious to consume a variety of foods with a resultant dietary restriction. There is also a relationship between disordered eating and psychological distress, associated with maladaptive coping mechanisms, depression, and stress [74,153].

In addition to disordered eating and adherence difficulties with gluten-free restriction, many studies have demonstrated that a GFD significantly increases the cost of food, with prices up to three times greater than for similar non-gluten-free products [154,155]. Gluten-free products are generally more expensive, and perceived costs remain a barrier to adherence [156]. There are many reasons that may contribute to difficulty with compliance of the GFD, including social exclusion, fewer food choices, accidental contamination, and cost.

## 10. Conclusions

Any individual on a restricted diet, whether avoiding lactose, gluten, or FODMAPs, should be screened for nutritional deficiencies. Patients on these diets have shown significant nutrient deficits that may require supplementation. Eliminating gluten in the diet is the treatment for GRDs and gluten sensitive IBS, although compliance with the GFD is challenging. Consequently, access to an educated dietician is essential to ensure a nutritionally balanced diet while being carefully monitored by a gastroenterologist to ensure long-term compliance. Furthermore, the dietary restriction used to treat IBS, lactose conditions, CD, and NCGS, can negatively impact quality of life. Therefore, medical providers managing and treating these conditions should be aware of the persistent challenging effects of nutrition on the overall health of these patients. As LI patients can tolerate small amounts of lactose, and NCGS and IBS patients have shown tolerance to some exposure to gluten, a reduced diet instead of strict avoidance may be adequately effective for symptom relief while preventing nutritional complications. Clinicians should consider a temporary restricted diet in these patients, with the goal of reducing symptoms followed by a gradual introduction of dairy or gluten products as tolerated. Subsequently, a prolonged dietary restriction may not be required for treatment in these conditions.

Future studies should examine the effects of diet reduction versus elimination in patients with LI, IBS, and NCGS. In addition, research should focus on the relationship between LI in adults and osteoporosis, as the relationship between low bone mineral density and osteoporosis remains unclear. There are conflicting studies on whether the FODMAPs diet positively affects the quality of life in IBS individuals; therefore, additional long-term examination of these patients is necessary. Furthermore, novel testing of GIP in the urine and stool has been used to detect gluten ingestion; however, the development of a reliable GIP control is necessary for the accurate evaluation of GFD adherence in GRDs. In addition, changes in the intestinal microbiome on a GFD have been reported, although additional longer studies are needed to assess the impact of this change on patients’ health. Conclusive data is essential for the study of the actual prevalence of IBS in patients with CD, for a better understanding of the relationship between these two conditions. Finally, more data is required to uncover the pathophysiology of NCGS and IBS and to establish the precise association between IBS and GRDs.

## Data Availability

Not applicable.

## Abbreviations

AGA: anti-gliadin antibody; CD, celiac disease; CDAT, Celiac Dietary Adherence Test; DGP, deamidated gliadin peptide; EMA, endomysial antibody; FODMAPs, fermentable oligo-, di-, and monosaccharides and polyols; GFD, gluten-free diet; GIP, gluten immunogenic peptide; GRDs, gluten-related disorders; HBT, hydrogen breath test; IBS, irritable bowel syndrome; IBS-D, irritable bowel syndrome diarrhea-predominant; IELs, intraepithelial lymphocytes; IgE, immunoglublin E; LI, lactose intolerance; LM, lactose malabsorption; NCGS, non-celiac gluten sensitivity; NRCD, nonresponsive celiac disease; TTG, tissue transglutaminase.
